# Sex‐specific natural selection on SNPs in *Silene latifolia*

**DOI:** 10.1002/evl3.283

**Published:** 2022-05-27

**Authors:** Lynda F. Delph, Keely E. Brown, Luis Diego Ríos, John K. Kelly

**Affiliations:** ^1^ Department of Biology Indiana University Bloomington Indiana USA; ^2^ Department of Ecology and Evolutionary Biology University of Kansas Lawrence Kansas USA

**Keywords:** dioecious, fitness, paternity, selection component analysis, sex‐specific selection, sexual dimorphism

## Abstract

Selection that acts in a sex‐specific manner causes the evolution of sexual dimorphism. Sex‐specific phenotypic selection has been demonstrated in many taxa and can be in the same direction in the two sexes (differing only in magnitude), limited to one sex, or in opposing directions (antagonistic). Attempts to detect the signal of sex‐specific selection from genomic data have confronted numerous difficulties. These challenges highlight the utility of “direct approaches,” in which fitness is predicted from individual genotype within each sex. Here, we directly measured selection on Single Nucleotide Polymorphisms (SNPs) in a natural population of the sexually dimorphic, dioecious plant, *Silene latifolia*. We measured flowering phenotypes, estimated fitness over one reproductive season, as well as survival to the next year, and genotyped all adults and a subset of their offspring for SNPs across the genome. We found that while phenotypic selection was congruent (fitness covaried similarly with flowering traits in both sexes), SNPs showed clear evidence for sex‐specific selection. SNP‐level selection was particularly strong in males and may involve an important gametic component (e.g., pollen competition). While the most significant SNPs under selection in males differed from those under selection in females, paternity selection showed a highly polygenic tradeoff with female survival. Alleles that increased male mating success tended to reduce female survival, indicating sexual antagonism at the genomic level. Perhaps most importantly, this experiment demonstrates that selection within natural populations can be strong enough to measure sex‐specific fitness effects of individual loci.

Males and females typically differ phenotypically, a phenomenon known as sexual dimorphism. These differences arise when selection on males differs from selection on females, either in magnitude or direction. Estimated relationships between traits and fitness indicate that sex‐specific selection is widespread, occurring in both plants and animals, and explains why so many species exhibit sexual dimorphism. Finding the specific loci experiencing sex‐specific selection is a challenging prospect but one worth undertaking given the extensive evolutionary consequences. Flowering plants with separate sexes are ideal organisms for such studies, given that the fitness of females can be estimated by counting the number of seeds they produce. Determination of fitness for males has been made easier as thousands of genetic markers can now be used to assign paternity to seeds. We undertook just such a study in *S. latifolia*, a short‐lived, herbaceous plant. We identified loci under sex‐specific selection in this species and found more loci affecting fitness in males than females. Importantly, loci with major effects on male fitness were distinct from the loci with major effects on females. We detected sexual antagonism only when considering the aggregate effect of many loci. Hence, even though males and females share the same genome, this does not necessarily impose a constraint on their independent evolution.

Sexual dimorphism is nearly universal in organisms with separate sexes and has evolved because males and females have different trait optima and, thus, experience sex‐specific selection (Lande 1980). There is abundant data on both plants and animals indicating that the strength and direction of selection on quantitative traits differ between the sexes (Arnqvist and Rowe 2005; Delph et al. 2011; Delph and Herlihy 2012). Evidence for sex‐specific selection on the loci that underpin trait variation is far more equivocal (Ruzicka et al. 2020). Sex‐specific selection can alter the overall strength of selection and, hence, the rate of adaptation. The form of sex‐specific selection can determine the level of genetic polymorphism, mutation load, local adaptation, and the location of polymorphic genes across the genome (Kidwell et al. 1977; Bull 1983; Rice 1984; Connallon and Knowles 2005; Foerster et al. 2007; Hedrick 2007; Otto et al. 2011; Connallon 2015; Grieshop et al. 2016; Svensson et al. 2018; Dapper and Wade 2020).

Quantitative genetic studies imply that selection on individual loci must differ between the sexes to some degree. If selection was uniform, the genetic variance in relative fitness would be the same in males and females, and the genetic correlation between sexes would be perfect (r_mf_ = 1; Lynch and Walsh (1998)). Refuting this, Chippindale et al. (2001) estimated that r_mf_ for the overall fitness of males and females was close to zero in *Drosophila melanogaster*, with positive correlations between some components (juvenile survival) canceled by negative correlations between other components (reproductive success of adult males and females). Connallon and Matthews (2019) provide a comprehensive review of r_mf_ estimates. At this point, it remains unclear whether sex differences in selection are typically incremental (the same allele is favored in both females and males but to differing degrees), or sex‐limited (fitness effects are limited to one sex), or antagonistic (the allele favored in males is detrimental to females).

After reviewing the many challenges in searching for sex‐specific selection on loci, Ruzicka et al. (2020) advocate for “direct approaches” where the fitness of individual males and females is predicted from their individual genotypes within natural populations. We here describe such an experiment, estimating the male and female fitness effects of Single Nucleotide Polymorphisms (SNPs) across the genome of *Silene latifolia*. This short‐lived, dioecious, herbaceous plant is sexually dimorphic for life‐history, physiology, and morphological traits (Delph 2007; Steven et al. 2007). We collected data on flowering and estimated female and male fitness in a full census of one population of *S. latifolia* from Virginia (USA). We genotyped field plants and their progeny using Multiplexed Shotgun Genotyping (MSG) RAD‐seq (Andolfatto et al. 2011). For female fitness, we counted all the seeds produced by each plant and determined if the maternal genotype at each SNP was associated with fecundity. Selection through differential male success was measured via two complimentary methods, paternity inference (Jones et al. 2010) and Selection Component Analysis (SCA; Christiansen and Frydenberg 1973; Monnahan et al. 2015), each of which is based on sequence data from a random collection of offspring of the female plants. Paternity inference uses the entire collection of SNP genotypes for each offspring to ascertain its father. The fitness measure is then the count of progeny sired by each male. SCA infers selection on SNPs without identifying the specific male that sired each offspring. Differential male success is inferred if allele frequencies in the population of successful male gametes–those that fertilize ovules to produce offspring–are different from allele frequencies in the entire population of males (Monnahan et al. 2021). This “male selection SCA” does not distinguish differential paternity by diploid males from subsequent gametic selection, that is, pollen competition and/or meiotic drive (Immler and Otto 2018; Beaudry et al. 2020). Thus, paternity selection is a subset of male selection. Finally, we also determined which plants survived to the start of the following reproductive season and if sex‐specific survival was related to genotype.

Sex‐specific selection at the phenotypic level has been thoroughly demonstrated in *S. latifolia* (Wright and Meagher 2004; Delph et al. 2011; Yu et al. 2011; Delph and Herlihy 2012). In this study, we determine whether flowering traits affect fitness measures similarly in males and females and whether fecundity and survival trade‐off with each other. Second, we identify specific polymorphisms contributing to male and female fitness variation, the effect of selection on these loci (measured as allele frequency change), and determine their pattern of sex specificity. We find that the partitioning of fitness into multiple components within each sex is essential to estimate genetic trade‐offs relevant to the maintenance of polymorphism.

## Methods

### STUDY POPULATION AND FIELD METHODS

**Figure 1:**
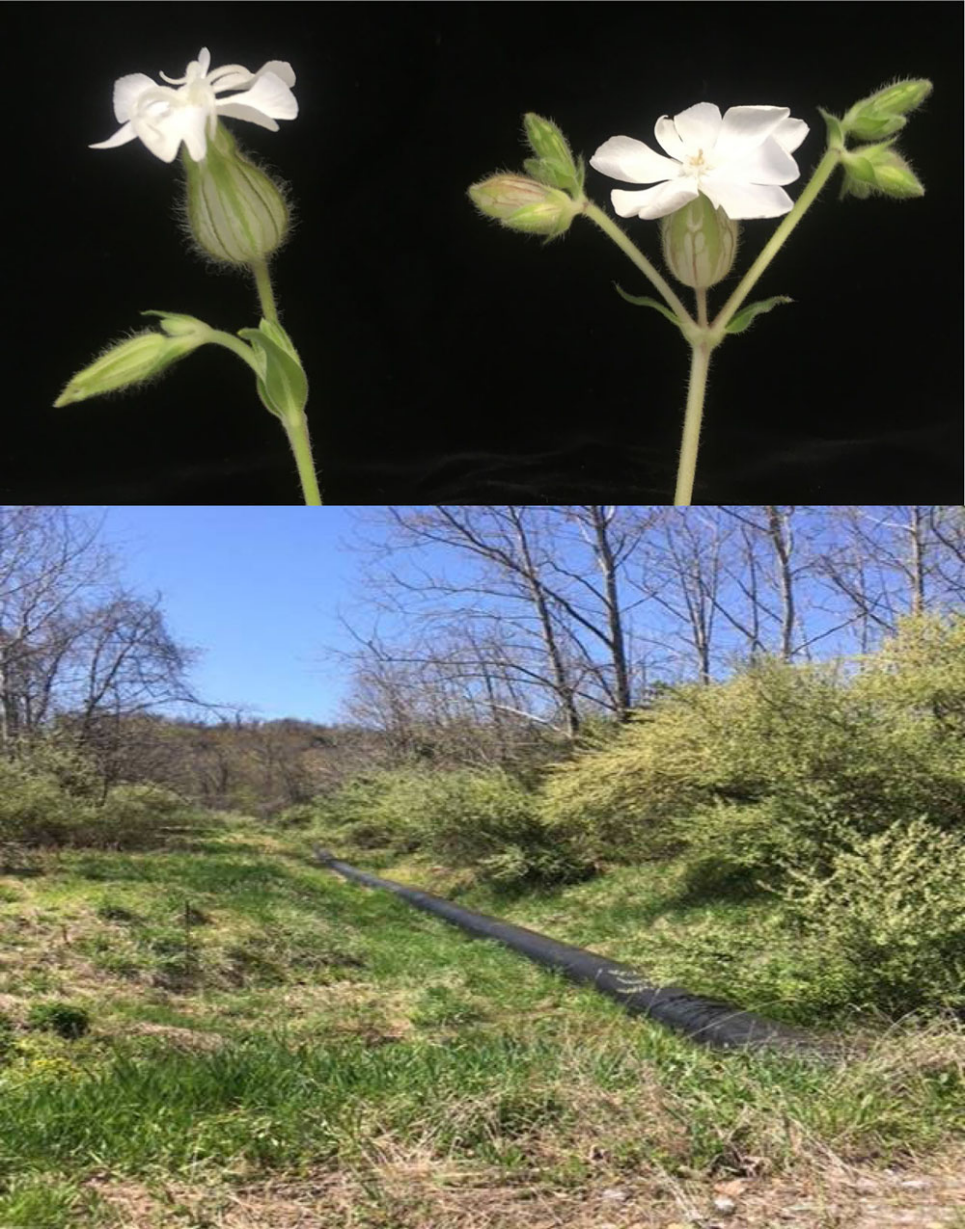
Top panel: *Silene latifolia* flowers from our study site: female (left) and male (right). Flowers open at dusk and are pollinated by night‐flying moths (Jürgens et al. 1996). Flowers on females have wider calyces, weigh more, and are fewer in number than those on males (Delph 2007). Bottom panel: view of the clearing in early spring 2018, showing the pipe that was laid in 2015.

In Western Virginia, *S. latifolia* routinely occurs in small patches in disturbed areas such as roadsides (Fields and Taylor 2014). Our study population is in a clearing (∼75 m long by 8‐m wide) associated with a drainage ditch running perpendicular to Norcross Road in Giles County, VA, USA (37.359913, −80.681412) (Fig. 1). Full details on this large, but recently derived population, as well as our field methods, are reported in Supporting information Section SA. Briefly, we marked every *S. latifolia* individual in the clearing (N = 1332) in early spring 2018 and collected leaf tissue for DNA extraction. A total of 41 of these plants never reached flowering in the season of study and are not considered further. For the remainder, we determined the sex of the plant based on flower morphology (Fig. 1), recorded the date of first flowering, and subsequently, the total number of flowers produced by each plant over the course of the season. Every fruit produced by females was collected, and we counted all the seeds. Thirty seeds from each female were retained for genotyping (see Results), with the rest distributed back into the study site near the maternal plant. In early spring 2019, we returned to the field site and determined which plants were still alive. Of the tagged plants, we were able to confidently score survival for 547 females and 470 males.

### DNA AND SEQUENCING

Using the Qiagen DNeasy Plant Mini Kit (Hilden, Germany), we extracted DNA from 1276 tissue‐collected field plants and 809 offsprings. The latter were a random selection of progeny (1–4 per maternal plant) grown in the University of Kansas greenhouse. We applied RAD genotyping using the MSG protocol (Andolfatto et al. 2011) with the restriction enzyme Csp6I (CviQI). RAD genotyping was employed because *Silene* has a large genome and this technique has proven effective by previous studies (e.g. Liu and Karrenberg 2018). We made replicate libraries for a subset of samples. The libraries were size selected for fragments 248−302 bp in length using a BluePippin (Sage Science). We sequenced 2500 DNA libraries (paired‐end sequencing, 150 bp on each end) using the Illumina NovaSeq6000 platform (S4 option, Novogene, Beijing).

### BIOINFORMATICS AND TESTS FOR SELECTION

We identified loci de novo using STACKs (Catchen et al. 2013). The catalog of loci was obtained after testing a series of different parameter options (Supporting information section SB describes the pipeline in detail). Genotype calling for SNPs within loci was executed with bwa/bcftools (Li and Durbin 2009) with subsequent filtering to eliminate SNPs that showed (a) deviations from Hardy–Weinberg equilibrium, (b) deviant mother–offspring transmission, (c) excessive divergence between males and females (see Results), or (d) a minor allele frequency less than 5%. After filtering, 55,145 SNPs remained. All these loci exhibit transmission patterns consistent with autosomal inheritance. Our ascertainment method did not identify sex‐linked loci.

Each SNP was used for paternity inference and tests of selection. We developed a genotyping model with SNP‐specific and plant‐specific error rates and fit the relevant parameters using maximum likelihood. For selection component tests, the key parameters in the model are the allele frequencies in each “cohort”: *q_M_* = frequency in males, *q_F_* = frequency in females, and *q_S_* = frequency in successful male gametes (those that successfully fertilize ovules to make seeds). SNPs exhibiting statistically significant divergence between *q_M_* and *q_F_* were suppressed. The selection component tests (Supporting information section SC) depend only on the genotype data and address the following questions: (1) Is *q_M_* different from *q_S_* indicating male selection? (2) Among males, does genotype affect survival into the next year? (3) Among females, does genotype affect survival into the next year? For each test, the direction and magnitude of selection is captured by Δ*q*, the predicted change in allele frequency.

Individual‐based tests for selection relate female genotypes to seed production and male genotypes to paternity. We estimated paternity by considering all SNP data from each mother–offspring pair, in relation to each male in the population. For each such “trio,” we calculated the log‐likelihood of the data, first assuming that the male is the sire and then treating it as a random sample from the population. A “paternity matrix” from these calculations was input to the Fractional Assignment of Paternity (FAPs) program (Ellis et al. 2018) for subsequent inference of the number of offspring sired by each male. While some mother–offspring pairs overwhelmingly favor a particular male as the sire, FAPs allow fractional assignments of paternity when no single male is indicated unambiguously. Paternity selection is assessed by relating the genotype likelihoods at each SNP for each male to the estimated number of offspring sired (Supporting information section SD). Female fecundity selection is estimated in two stages: genotype can affect the probability that a plant makes seeds at all, and second, the average seed production given that it produces seed. The two affects are aggregated to obtain Δ*q* for differential female fecundity. We developed a permutation scheme to test whether SNP‐specific ∆*q* differed from zero by either paternity or female fecundity selection (Supporting information Section SD, Supporting information Fig. S1).

We compared different selection component estimates on the same SNP using the Spearman rank correlation (*ρ*) on component specific Δ*q* values. Here, we also applied a permutation method (Supporting information section SE) to establish significant associations. Standard parametric tests, performed with JMP Pro 15 (SAS Institute), were used to test for phenotype effects on male and female fitness measures (Supporting information section SF). Finally, calculations of linkage disequilibria (LD) among SNPs are described in Supporting information section SG. All original programs used for these analyses are given in Supporting information File S1 with instructions on how to operate the entire pipeline. False discovery rates (FDRs) from *p*‐values of tests were calculated using the padjust() function in R.

## Results

### RELATIONSHIPS BETWEEN TRAITS AND FITNESS

**Figure 2:**
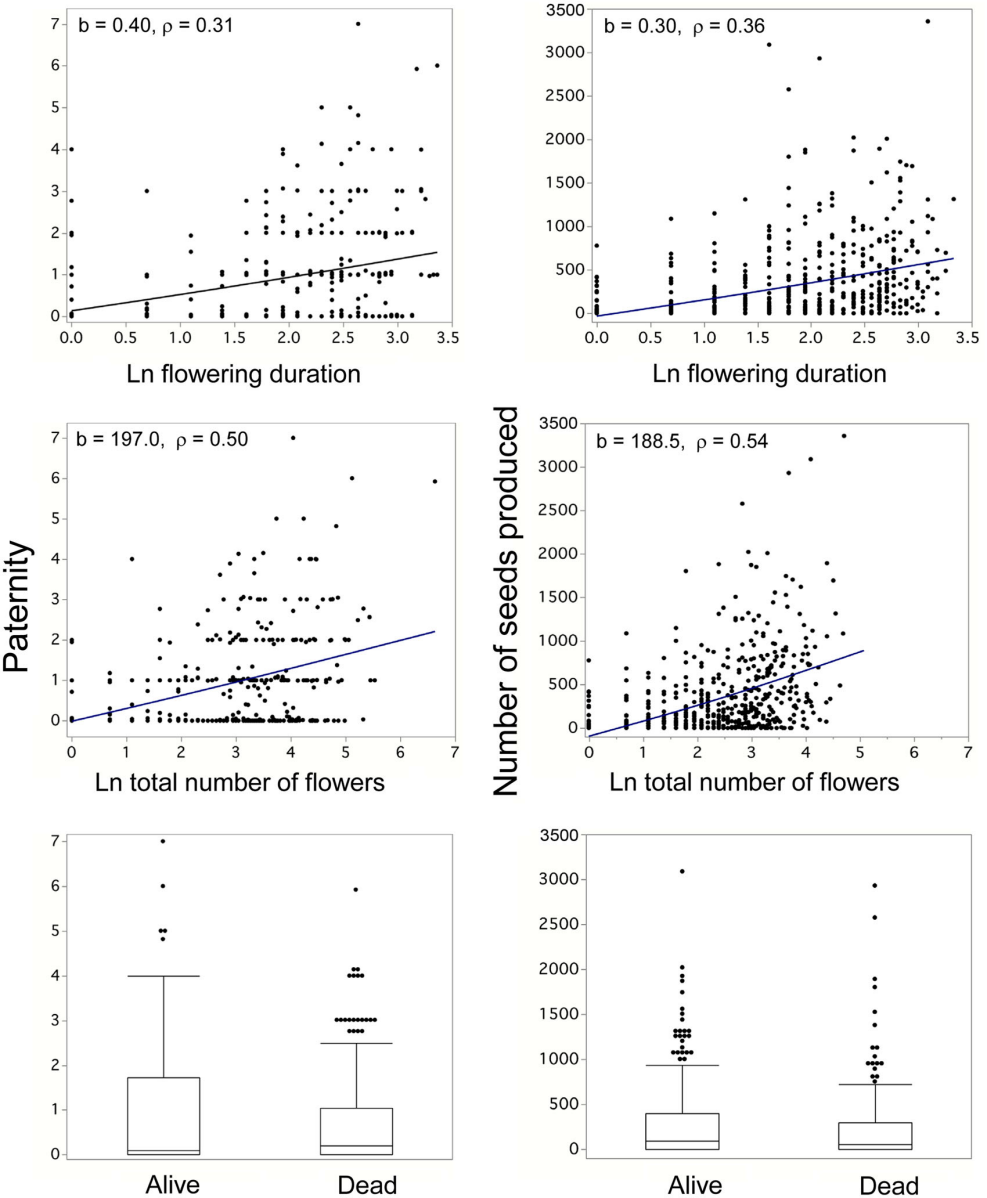
The relationship between our two fitness measures (paternity [number of offspring sired by males] and the number of seeds produced [females]), two flowering traits, and survival. Top panel: natural log of the number of days each plant flowered in 2018. Middle panel: natural log of the total number of flowers produced in 2018. The linear regression slopes (b) and Spearman correlations (ρ) are reported for each contrast. Bottom panel: the state of each plant in 2019 (box plots show the medium value, the first and third quartile, the whiskers, and outliers). The point for one female plant is not shown (although the data were included for analyses); over a 22‐day period (Ln value = 3.09), this plant, which was alive in 2019, produced 8539 seeds from 160 flowers (Ln value = 5.08).

In 2018, the flowering population was significantly female biased (697 females, 594 males [54% females], *χ* ^2^ = 8.2, *p* = 0.004). Female fecundity and male siring exhibited right‐skewed distributions, although much more so for females (Supporting information Fig. S2). The sexes were significantly sexually dimorphic for flowering traits. Males flowered for more days (least square means (SE) of natural log of flowering duration = 1.9 [0.04] versus 1.6 [0.03], males versus females, respectively; *t* = 5.36, *p* < 0.0001) and made more flowers than females (natural log of the total number of flowers produced = 2.9 [0.05] vs. 2.1 [0.05]; *t* = 10.75, *p* < 0.0001). The fitness of males and females covaried with flowering traits, consistent with concordant selection via male and female fitness at the trait level. For males, paternity increased significantly with flowering duration (F_1,469_ = 42.5, *p* < 0.0001, *R* ^2^ = 0.08) and total flower number (F_1,469_ = 64.4, *p* < 0.0001, *R* ^2^ = 0.12; Fig. 2). The same was true for the total number of seeds produced by females (flowering duration‐F_1,676_ = 83.1, *p* < 0.0001, *R* ^2^ = 0.11; total flower number‐F_1,679_ = 143.3, *p* < 0.0001, *R* ^2^ = 0.17; Fig. 2). Each of these associations remain highly significant (*P* < 0.001) if evaluated with a nonparametric Spearman Rank correlation (*ρ*). In 2019, we were able to locate and determine the survival for 547 females and 470 males. Significantly more males died than females (54 vs. 38%, respectively; *Z* = −5.14, *p* < 0.0001). Survival was not significantly related to paternity of males (*χ* ^2^ = 0.57, *p* = 0.45) or the number of seeds produced by females (*χ* ^2^ = 1.99, *p* = 0.16) in the previous year (Fig. 2).

### SNPs ASSOCIATED WITH FITNESS

We performed five tests for selection on the aggregate of plant and genotype data. We tested whether plant genotype affects the fecundity of females and the subsequent survival of both males and females into the next year. We next tested for selection through differential male reproductive success, first via siring as a strict function of the diploid male genotype (paternity) and then through the combined action of haploid and diploid selection (male selection). Each selection test was applied to 55,145 autosomal SNPs that passed filters. In terms of the strength of evidence for selection on individual SNPs, there was a clear ordering of processes: male selection > paternity selection > female fecundity > male survival > female survival.

For each test, selection is reported as the predicted allele frequency change (Δ*q*) generated by that form of selection. Male selection was evident across the genome: 1519 SNPs (2.75%) produced Δ*q* passing our genome‐wide significance threshold: FDR <0.1 (Benjamini and Hochberg 1995). There were 12,065 SNPs with an FDR <0.5. Male selection showed a clear tendency to favor the less common base: Δ*q* for the minor base was positive for 1351 of 1519 SNPs (Supporting information Fig. S3). Across all SNPs (significant and not), there is a slight but significant tendency for the minor base to be more frequent in successful male gametes than in the entire adult male population. In contrast to male selection, the other two selection component tests (male and female survival to the next year) produced much weaker evidence for SNP‐specific selection. There were no tests with an FDR <0.1 within either sex. A total of 30 SNPs had FDR <0.5 for male survival, six for female survival.

Paternity assignment was imperfect owing to highly variable sequencing depth among samples. We evaluated only 481 of 594 males, because 113 were not sequenced sufficiently for inference. We were able to confidently identify the sire for 340 offsprings, but with fractional assignment (Ellis et al. 2018), attributed paternity to 418 of 809 offspring. Using fractional paternity as a fitness measure, 53 SNPs (1% of total) produced FDR <0.1 for paternity selection. As with male selection, paternity selection favored the less common base (52 of 53 cases). Importantly, the overall signal for paternity selection is wider than the 53 genome‐wide significant SNPs. A total of 3271 SNPs have FDR <0.5, suggesting that over a thousand SNPs were affected by differential paternity. Significant tests for selection through differential female fecundity (seed production) were much less common than for paternity: 13 SNPs (0.02% of total) with an FDR <0.1, 165 with an FDR <0.5.

**Figure 3:**
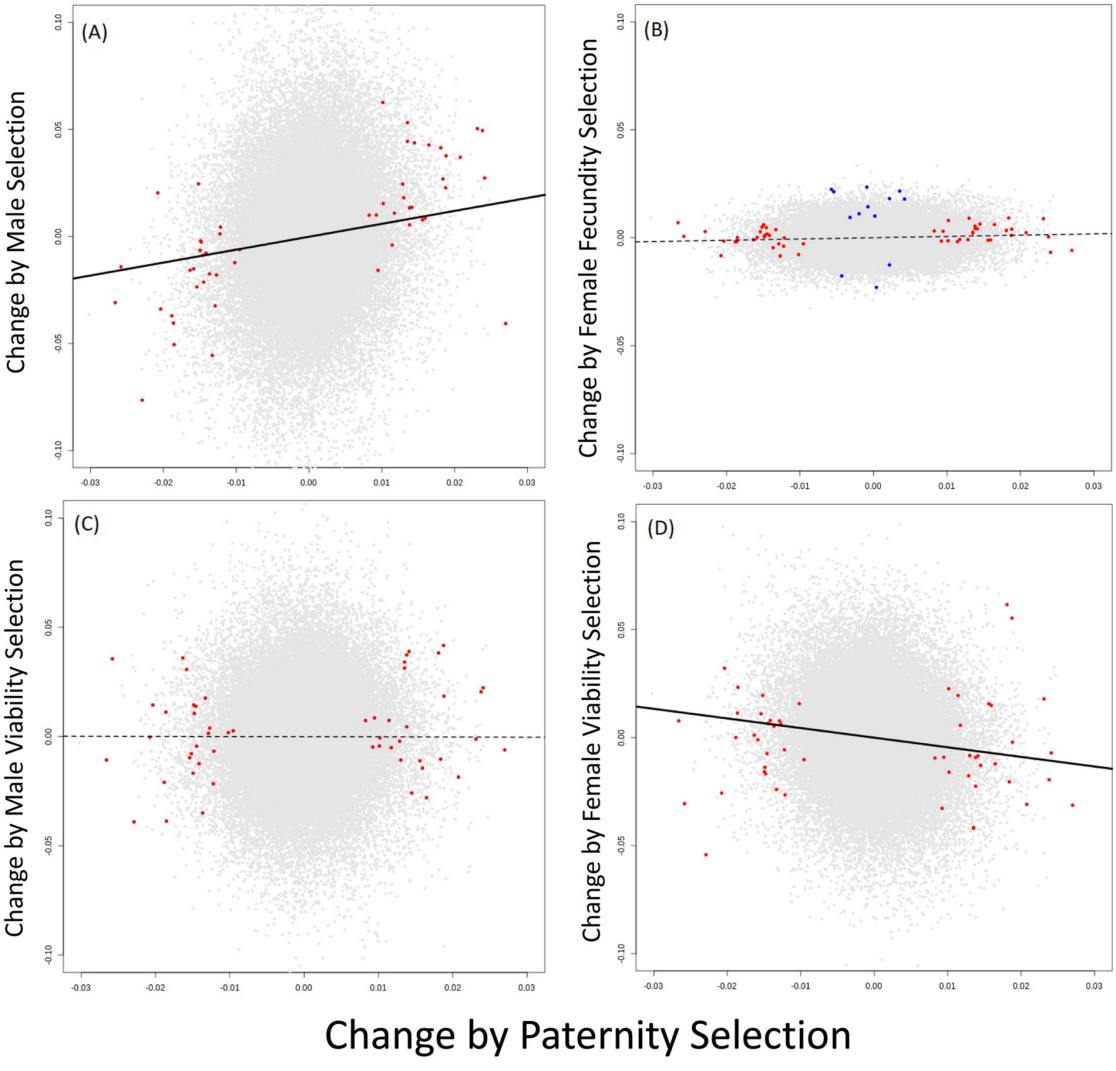
The relationship between Δ*q* from paternity selection and the other four components of selection: (A) male selection, (B) Female fecundity selection, (C) male survival, and (D) female survival. Red points denote the 53 SNPs that were genome‐wide significant (FDR < 0.1) for paternity selection (jiggered to show all 53 SNPs). In (B), blue points are the 13 SNPs significant for seed production in females. The lines were calculated by least squares but association tests on all SNPs were based on permutation using the Spearman correlation as a test statistic. The solid lines (A and D) denote statistically significant associations.

We cannot determine the number of distinct loci that are targets of selection, as opposed to hitchhikers (Maynard Smith and Haigh 1974), owing to possible LD among SNPs. Our genotyping method does not provide haplotypes, but we can coarsely estimate inter‐SNP associations from the covariance of diploid genotype scores (Rogers and Huff 2009). The average SNP showed minimal association with >99% of the genome, but moderate to strong association with the remaining 0.1% of SNPs (Supporting information Fig. S4). The 53 paternity‐significant SNPs (Fig. 3A) corresponded to 39 distinct “loci” with an average *r* ^2^ = 0.99 between SNPs within loci and an average *r* ^2^ = 0.02 between loci. There were nine distinct loci for the 13 significant SNPs for female fecundity (average *r* ^2^ = 0.995 and 0.047 within and between loci, respectively). In some cases, SNPs within loci were from the same RADtag and, thus, closely linked. However, we could not estimate LD as a function of larger inter‐SNP distances because the chromosomal locations of our RADtags are unknown.

The question of sex specificity is addressed by contrasts between different selection estimates on the same SNPs. We estimated associations using the Spearman rank correlation statistic (*ρ*) between Δ*q* estimates from different selection components (Supporting information Methods Section SE). We obtained a null distribution for *ρ* (for each pair of selection components) by permuting fitness measures within sexes (paternity and survivorship within males, seed production, and survivorship within females), recalculating all Δ*q* at each of the 55,145 SNPs, and then calculating each *ρ* from the resulting data. This method preserves associations among loci and is, thus, robust to nonindependence of tests owing to LD. Two contrasts proved significant when compared to 5000 permuted datasets: paternity selection was positively correlated with male selection (*ρ* = 0.11, *p* < 0.004), but negatively correlated with female viability selection (*ρ* = −0.10, *p* < 0.013).

Alleles favored by paternity were also favored by male selection (Fig. 3A), which is expected given that the former is a component of the latter. The magnitude of the correlation is not large (*ρ* = 0.11) but this is inevitable given that we are considering all SNPs and most are not likely affected by either selection component. These neutral SNPs reduce the correlation by contributing noise centered on the origin (Δ*q_x_* = 0, Δ*q_y_* = 0). If we limit consideration to SNPs significant for paternity selection (red points in Fig. 3), the correlation becomes much stronger (*ρ* = 0.68). In contrast, there is no apparent association of paternity selection with either female fecundity (Fig. 3B) or male viability (Fig. 3C). The lack of correlation between male paternity and female fecundity is reinforced by the observation that no SNPs were significant for both paternity and female fecundity at FDR <0.1. If we step back to accept SNPs with an FDR <0.5 for either component, six SNPs were significant for both paternity and female fecundity, but that is fewer than expected by chance (9.8). Finally, alleles favored by paternity selection tended to reduce female survival (Fig. 3D). The negative correlation here (*ρ* = −0.10) must be driven by a highly polygenic response. It cannot be explained by a small subset of SNPs with strong effects on either component. No SNPs had FRD <0.1 for female viability, and the paternity significant SNPs are not strong predictors of female viability (Fig. 3D, red points).

## Discussion

The strength and direction of natural selection routinely differ between males and females. The consequences of this sex‐specific selection depend on whether trait expression in males is determined by the same genetic loci as in females. We investigated a natural population of the dioecious plant *S. latifolia* to determine how male and female fitness were related to flowering traits and whether SNPs affecting fitness were shared between the sexes. We found that two highly sexually dimorphic traits, flowering duration and flower production, were under concordant, positive selection in both sexes. However, beyond phenotypes, we found evidence for sex‐specific selection on individual loci, especially in males. We found almost no overlap between individual SNPs affecting male and female fitness. However, we did detect sexual antagonism when all SNPs were considered (Fig. 3D) suggesting a highly polygenic tradeoff between paternity and female viability.

Recent works on genomic signals of sex‐specific selection have focused on allele frequency differences between sexes (Cheng and Kirkpatrick 2016; Lucotte et al. 2016). Differences are predicted at SNPs with conflicting effects on male and female viability. In this study, we suppressed all SNPs with even a marginally significant (*p* < 0.05) difference in allele frequency between adult males and females. We adopted this conservative procedure to avoid spurious results owing to mismapping of reads from sex chromosomes to autosomal RAD tags (Bissegger et al. 2020; Kasimatis et al. 2021). *Silene latifolia* may be particularly prone to this kind of error given the recent evolution of its sex chromosomes (Kasimatis et al. 2021). The direct prediction of fitness measurements from individual genotypes avoids this potential bias (Ruzicka et al. 2020). This approach succeeded in that we were able to find SNPs that strongly affected fitness (Fig. 3) despite that preselection genotype frequencies were similar in males and females. Admittedly, by suppressing SNPs that differed between adult males and females, we may have missed some targets of selection, that is, SNPs with viability effects that differ strongly between males and females (Ruzicka et al. 2020). We tested 3200 SNPs that were filtered owing to male–female divergence for effects on survivorship into the next year (2019, Fig. 2, lower panel). These male–female divergent SNPs showed no more evidence of effects on survivorship into 2019 than the main set of 55,145 SNPs that passed filters.

### TRAIT–FITNESS RELATIONSHIPS

We found that both male and female fitness increased with increasing duration of flowering and flower production (Fig. 2). These two higher‐level phenotypes, which result from the interplay of many traits, were sexually dimorphic. Flower production has been shown to be the most highly sexually dimorphic trait of 28 traits investigated in *S. latifolia* (see review in Delph 2007). Common garden, quantitative genetic, and artificial‐selection studies have also shown that variation in flower production has a genetic basis, a positive between‐sex correlation, is genetically correlated with many other sexually dimorphic traits, and trades‐off with flower size (Meagher 1992, 1994; Delph et al. 2004; Steven et al. 2007; Delph and Bell 2008).

In a previous multiyear field study of *S. latifolia*, Delph and Herlihy (2012) showed that sexual selection favored males that made small flowers, as these males made many flowers early in the season, which corresponded with the time of high seed production by females. Seed production was higher in females that made relatively large flowers, and hence, relatively few flowers. Both males and females that flowered extensively paid a cost in terms of longevity. Hence, the different forms of selection—sexual, fecundity, and viability—were in opposition in the males, but not in the females (sexual and fecundity selection favored males that made many small flowers, and viability selection favored males that made relatively few flowers). Another study showed that viability selection favors males with thick leaves under dry conditions (i.e., selection was condition dependent), whereas females experience weakly positive or significant stabilizing selection on the same trait (Delph et al. 2011). These sex‐specific estimates for phenotypic selection go a long way toward explaining why the sexes are sexually dimorphic, especially for flower number. In the present study, we did not see striking differences between how male and female flowering phenotypes related to reproductive success (Fig. 2), but there was clear evidence of sex‐specific selection occurring at the SNP level (Fig. 3). This difference may simply reflect the fact that many of the SNP effects on fitness were mediated through traits that we did not measure.

### SEX‐SPECIFIC SELECTION ON SNPs

We found a pattern of sex‐specific selection at individual SNPs, with distinct sets of loci contributing to male and female fitness variation (Fig. 3). This outcome could be anticipated from previous results showing that sex‐specific QTL explain a significantly greater percent of the variation in sexually dimorphic traits than loci affecting the traits in both sexes (Delph et al. 2010). Several large‐effect QTLs have been found to only affect fitness‐related traits when present on the Y chromosome (Scotti and Delph 2006). The aggregate effect of QTLs to patterns of intrapopulation variation is measured by their contribution to genetic (co)variance, or “**G**” matrix (Kelly 2009). Steven et al. (2007) estimated the **G** matrix for each sex of *S. latifolia* and noted a lack of shared structure between sexes suggesting extensive sex‐limited genetic effects on traits. The aggregation of results from SNP‐level, QTL‐level, and **G** matrix studies show that sex differences in the inheritance of fitness variation are congruent with theory on the resolution of sexual conflict (Lande 1980; Rice 1984; Rhen 2000).

We obtained much stronger evidence for selection on males than females, both from the male selection component test and differential paternity. Statistical power does not explain the male/female difference. Sample size was larger for females than males and we estimated female fecundity with much greater precision. For each female, we obtained a full count of seeds produced, whereas paternity was assigned for only a tiny fraction of the 194,437 seeds made within the population. A simple explanation for the stronger signal is simply that there is more genetic variation for fitness in males than females. As has been shown for animals (Bonduriansky et al. 2008), *S. latifolia* males may be living life more “on the edge” because of conflicting selection pressures (sexual vs. viability selection; see above). Here, greater genetic integration may allow the retention of genetic variation (see also Rowe and Houle 1996). The **G** matrix of males has been shown to contain stronger and more trait–trait correlations than that of females (Steven et al. 2007). In addition, more QTL co‐occur in males than females (Delph et al. 2010). Greater selection on males compared to females is also congruent with a study that showed that Qst/Fst ratios are an order of magnitude higher for males than females for flower size (which trades off with flower number; Yu et al. 2011).

### MALE VERSUS PATERNITY SELECTION

Between our two male‐specific tests, a much larger number of SNPs were significant for male selection than for paternity selection. This likely has both biological and statistical causes. Male selection is the combined effect of differential siring by diploid males and gametophytic selection (pollen competition), processes that generate allele frequency differences between adult males and pollen grains that successfully fertilize seeds. A large fraction of the plant genome is expressed in haploid pollen (Bernasconi et al. 2004; Borg et al. 2009) providing ample opportunity for gametic selection (Delph 2019; Tonnabel et al. 2021). Strong selection for haploid beneficial alleles has been shown to occur in plants as a consequence of their not being masked by their sister alleles (Arunkumar et al. 2013). Evidence for selection via pollen competition specifically in *S. latifolia* comes from the rate of degeneration of Y‐linked genes. The rate of decay is slower than expected based on the age of the sex chromosomes (Bergero and Charlesworth 2011; Chibalina and Filatov 2011; Krasovec et al. 2018), consistent with the idea that degeneration is slowed because they are important during pollen competition (Bull 1983).

A simple feature of our results supports paternity selection as a subset of male selection. Paternity selection SNPs nearly always showed a significant effect through male selection and these effects were in the same direction (Fig. 3A). The reverse is not true–many SNPs significant for male selection showed no effect on paternity. Additionally, it may sometimes be easier to detect differential paternity through the male selection component test (SCA). The SCA absorbs the signal of paternity selection both from unassigned siring events as well as from those offspring where we could identify the father. The paternity test is limited to data from the latter category. In this study, we were unable to assign paternity for nearly half the genotyped offspring. In fact, it is possible that some of our offspring were sired by males outside our delimited population. Allele frequency change owing to pollen immigration can affect the male SCA test. If the donor population is divergent in allele frequencies, immigration will have a genome‐wide effect (Monnahan et al. 2015). The fact that we here see a very slight inflation of the minor allele in the mean Δ*q* from male selection (across all SNPs) is consistent with an immigration effect.

An unexpected observation from this study was the general tendency for selection (male or female) to favor the less common base at significant SNPs. With constant selection, we expect the favored allele to become more common than the deleterious allele over time. With temporally fluctuating selection, however, there is no clear expectation regarding allele frequency and direction of selection within a single generation. Scotti and Delph (2006) and Delph and Herlihy (2012) hypothesized that balancing selection acts on alleles in males of *S. latifolia* owing to environmental heterogeneity. In favorable conditions, alleles conferring a mating advantage will increase, while alternative alleles are favored in more stressful environments (where viability selection becomes more important). Measurement of environmentally driven changes in SNP‐fitness associations through time could test this hypothesis (e.g., Troth et al. 2018; Machado et al. 2021).

Rare alleles may also become favorable if a population experiences a major environmental shift or invades a novel environment. In fact, our study population was recently established in a novel habitat. The habitat was created in 2015 by the removal of vegetation, digging of a long trench, followed by the laying of a drainage pipe along the length of the trench (Fig. 1). We surveyed the population in 2016 and estimated the population size at that time to be 200–300 plants. It had expanded dramatically in size (N > 1300 in 2018) by the time we measured plants for this experiment, with a substantial portion of the plants colonizing the sides or bottom of the trench. We do not know the exact physical conditions experienced by the ancestral population (or populations), that is, those plants whose propagules established our “pipe population.” However, the light, temperature, and moisture regime within the trench are notably different than the roadside patches, where *S. latifolia* typically occurs in this area.

## Conclusions

This experiment provides evidence that the genetic variants affecting male and female fitness are largely independent, with some indication of a negative genetic correlation between paternity in males and survivorship of females (Fig. 3D). We hypothesize that this disconnect is more a consequence of selection than mutation. Mutation‐accumulation experiments in *Drosophila* suggest that most new mutations will have similar effects in each sex (Mallet et al. 2011; Sharp and Agrawal. 2013; Allen et al. 2017). Mutations with sex‐limited or sexually conflicting effects may persist as polymorphic for longer than mutations with sexually consistent effects if the latter are rapidly fixed or are lost from a population. Relevant to this argument, our experiment considered only mutations with a minor allele frequency of at least 5%. Mutations that reduce fitness in both sexes may segregate at low frequencies and would have been excluded from our analyses. While limited to intermediate frequency polymorphisms, the results do provide clear “proof‐of‐concept” at least for this class of variants. We found that the process of natural selection was strong enough to measure sex‐specific fitness effects individual loci—an essential requirement for success of the direct method of study to sex‐specific selection (Ruzicka et al. 2020).

## AUTHOR CONTRIBUTIONS

LFD and JKK conceived of and designed the study. LDR, LFD, and JKK contributed to the field component in 2018. LFD and LDR surveyed survivorship in 2019. KEB grew up progeny and performed the genotyping. JKK developed the bioinformatics pipeline and the SNP‐level tests for selection. LFD conducted trait‐based statistical analyses and JKK conducted all genomic analyses. JKK and LFD drafted the manuscript, and all authors contributed to and/or approved the final version.

## DATA ARCHIVING

The data associated with this manuscript will be archived prior to the time of publication.

Associate Editor: S. Wright

## Supporting information

Supplemental Methods

**Figure S1**. We use permutation to establish significance levels for SNPs affecting paternity and seed set.
**Figure S2**. The distribution of seeds per female (top panel, n = 687) and offspring sired per male (lower panel, n = 481)
**Figure S3**. The predicted change in allele frequency (minor base) owing to Male selection is reported for SNP with significant tests (Sig means FDR0.1)
**Figure S4**. The density function for LD among SNPs estimated from 1000 randomly selected SNPs (of the 55,145 total) contrasted all other SNPs.
**Figure S5**. The relationship between heterozygosity and number of called SNPs is depicted for the final genotype calls.

Supplementary material
