# Supplementary material for: Sex‐specific natural selection on SNPs in Silene latifolia
Source: Evol Lett. 2022 May 27;6(4):308–18. doi: 10.1002/evl3.283 (PMC9346077; doi:10.1002/evl3.283)
Supplement: Supplementary file 1 — Supplemental Methods [file EVL3-6-308-s002.docx]

Sex-limited natural selection on SNPs in *Silene latifolia*

SUPPLEMENTAL METHODS

**A. Study population and field methods**

The population occupies a clearing surrounding an industrial pipe. The habitat was highly variable along its width, with some areas receiving full sun and others shaded by trees and shrubs. In 2015, a pipe was laid in the clearing, creating a large disturbance event. Three hundred *S. latifolia* individuals were estimated to be in the clearing the summer following this event. This number had increased over 4-fold two years later. The population would have contained individuals of different ages. In early spring 2018, every *S. latifolia* individual in the clearing was marked by a numbered tag held in place with a nail pushed into the ground, and its spatial coordinates were recorded. Leaf tissue was taken from each plant and dried in silica gel for DNA extraction. The date upon which each individual opened their first flower, which ranged from 26 April to 27 July, was recorded. Once a plant began flowering, the number of new open flowers was counted in the early morning every other day throughout the flowering season. Because flowers on males last two days before dropping off and two-day old flowers on females can be distinguished from newly open flowers, this allowed us to determine both flowering duration and the total number of flowers produced per plant. Flowering duration and flower number are both higher-level phenotypes, ones for which many biological components contribute. Every fruit produced by females was collected into a paper envelope when seeds were mature, but prior to seed dispersal, and the seeds were subsequently counted. Thirty seeds from each female were retained for paternity analysis, and all other seeds were distributed back into the study site near the maternal plant. In early spring 2019, we returned to the field site and determined which plants were still alive. Of the tagged plants, we were able to confidently identify and record survival (dead/alive) for all but 274, based on the combination of the presence of the tag and mapped coordinates for each plant.

**B. Theory and bioinformatics of genotype calling**

We initially attempted to score SNPs by mapping reads to the Silene draft genome (Papadopulos et al. 2015), but obtained few loci that passed subsequent filters (described below). We then used STACKs (Catchen et al. 2013) to obtain loci de novo (loci are hereafter called RADtags). To obtain the catalog of RADtags, we applied STACKs to sequence data from 20 high quality female samples because the total volume of data is too large to use STACKs on all samples. Each end of a read-pair is treated as a distinct sequence (pairing is ignored for locus identification). We constructed catalogs using a range of parameter settings (M=2/n=1; M=3/n=2; M=4/n=3; and M=5/n=4), creating a catalog for each. We then mapped the sequence data from 200 males and 200 females (all with at least moderate sequencing depth) to each catalog using bwa mem. We then used bcftools mpileup to call SNPs on these 400 individuals (Li and Durbin 2009). The vcf files from each of the four parameter settings were then thinned to bi-allelic SNPs called in at least 150 of 400 samples (minimum depth of 5 for calls), with a minimum mapping quality of 40, and with an estimated minor allele frequency greater than 0.05. We determined genotype frequencies in each sex and classified SNP as “balanced” (heterozygotes about equally frequent in males and females), “male deficient” (heterozygotes under-represented in males), or “female deficient.” This balanced SNP set was retained and used to evaluate each catalog for quality of calls with subsequent analyses. The used the programs Silene.400.p1.py and Silene.400.p2.py in sequence on each of the four vcf files. These programs, and those cited below, were written in Python 2.7 and are contained in Supplemental File 1.

Next, we mapped the full collection of 2500 samples to each of the four catalogs and called SNPs using bwa/bcftools. We tested genotype frequencies at each SNP retained from the preceding analysis of 400 plants against Hardy-Weinberg expectations. We suppressed SNPs with fewer than 150 called individuals and those that showed a significant excess of heterozygosity (X^2^ > 5.99, p < 0.01) as this likely reflects badly constructed loci. We retained, at this stage, loci with under-calling of heterozygotes as this is a common feature of MSG data and can be addressed in the genotyping model. For this, we determined the ratio of observed to expected heterozygosity for each sample and then compared this to the number of SNPs scored for that sample. We found a positive relationship (Supplemental Figure S5). A relationship between quality of data (as measured by number of SNPs scored in the sample) and the probability that a heterozygote reports both alleles has been shown previously in MSG studies of numerous plant species (Monnahan et al. 2015; Wessinger et al. 2018). These calculations were executed using Silene.full.vcf.p1.py and then Silene.select.p1.py.

At a SNP, the VCF file reports the genotype call for a plant as 0 (reference allele homozygote), 1 (heterozygote) or 2 (alternative homozygote). We express the likelihood of each of these observed genotypes (OG) as a function of the true genotype (TG) as:

$P\left[ OG=0 | TG=RR \right]=1-e_{1}-e_{2}$

$P\left[ OG=0 | TG=RA \right]=e_{1}$

$P\left[ OG=0 | TG=AA \right]=e_{2}$

$P\left[ OG=2 | TG=AA \right]=1-e_{1}-e_{2}$

$P\left[ OG=2 | TG=RA \right]=e_{1}$

$P\left[ OG=2 | TG=RR \right]=e_{2}$

$P\left[ OG=1 | TG=AA \right]=\frac{1}{2}\left( 1-h\left( 1-e_{3} \right) \right)$

$P\left[ OG=1 | TG=RA \right]=h\left( 1-e_{3} \right)$

$P\left[ OG=1 | TG=RR \right]=\frac{1}{2}\left( 1-h\left( 1-e_{3} \right) \right)$

eqs S1

The $e_{x}$ terms are SNP-specific error rates: $e_{1}$= probability that a homozygote appears to be a heterozygote, $e_{2}$=probability that a homozygote appears to be the alternative homozygote, $e_{3}$= probability that a heterozygote appears to be a homozygote. In the last case, we assume the error to either homozygote is equally likely. The quantity *h* is the plant-specific probability that a true heterozygote reports both alleles (and thus gives OG = 1). We estimate this value from the heterozygosity versus calls-per-plant relationship (Figure S7), with *h* near 0.5 for plants with very weak data increasing to 1.0 for plants with more complete coverage.

Given the family structured plants (females with progeny) along with males, we calculated the likelihood for the entire dataset for each SNP as a function of the error rates in eqs S1 and up to four distinct allele frequencies: $q_{M}$= frequency in males, $q_{F}$= frequency in females, and $q_{S}$= frequency in successful male gametes (those that sire offspring). In our initial run (before culling loci as described below), we estimated a single q ($q_{M}$= $q_{F}$= $q_{S}$) and constrained $e_{x}$ to a maximum value of 0.02. We maximized likelihoods using the bfgs algorithm as implemented in Scipy (Virtanen et al. 2020). The program Silene.select.p4.py conducts model fitting for eqs (1) at every SNP and, given the MLE for parameters, Silene.select.p5.py outputs genotype likelihoods for each individual at each SNP.

After estimating allele frequency and calculating genotype likelihoods at each SNP, we applied AYMMr.py to identify loci with aberrant inheritance. We accepted SNPs only if Δ(mom)>1 and Δ(notma)<0. The pipeline up to this point was applied to all plants for each of the four Stacks catalogs. At this stage, we identified the M4/n3 catalog as optimal. The fraction of SNPs passing the AYMMr filter for M4/n3 was substantially higher than for M5/n4. This fraction was very slightly lower than for M3/n2, but the number of good SNPs for M4/n3 was much greater than M3/n2. The M4/n3 catalog was thus chosen as the ‘reference genome’ and used for all subsequent analyses. Given the more limited collection of 60,880 SNPs that survived filters, we re-estimated the heterozygosity versus called loci relationship (slightly updated h values for each plant) and then the SNP specific error rate and allele frequency parameters (reapplication of Silene.select.p1.py, Silene.select.p4.py, and Silene.select.p5.py).

**C. Selection component tests and Paternity inference**

The first set of selection tests are a function of only the genotype data. We performed a series of selection component tests by obtaining the maximum likelihood of the data with allele frequencies entirely unconstrained ($q_{M}\neq$ $q_{F}\neq$ $q_{S}$) to constrained models with fewer than 3 free parameters. Male selection is indicated if the likelihood of the general model is much higher than a constrained model where $q_{M}=$ $q_{S}$). A significant difference between $q_{M}$ and $q_{F}$(null model constrains $q_{M}=$ $q_{F}$) can be caused by sex-specific viability selection. However, for reasons discussed above, we suppressed all SNPs that produced even a marginally significant test for male/female divergence, leaving 55145 SNPs for the 5 remaining tests. For males and females separately, we tested whether allele frequency differed between plants that survived to the next year or not (survival test). For each test, the direction and magnitude of selection is captured by ∆q, the predicted change in allele frequency: ${\Delta q}_{male selection}=(q_{S}-q_{M})/2$, ${\Delta q}_{male viability}=(q_{M,alive}-q_{M,dead})/2$, and ${\Delta q}_{female viability}=(q_{F,alive}-q_{F,dead})/2$. The halving owes to the fact that selection is limited to one sex, which also applies to the paternity and female fecundity $\Delta q$ described below. The selection component tests were executed, one SNP at a time, using python programs. The program GSCA.Silene.py to estimates $q_{M},$ $q_{F}, and$ $q_{S}$ and perform the Likelihood Ratio Test for male selection. Next, we ran Silene.select.GL.py to determine the genotype likelihoods for all male and female plants (the information required for the remaining tests). Next, we ran Silene.GL.maleviability.py and Silene.GL.fem.viability.py to test for viability selection in males and females, respectively.

The remaining two tests relate the inferred SNP genotypes of adult males to estimated paternity (Paternity selection test) and the genotypes of adult females to seed set (Female fecundity test). We estimated paternity by considering all SNP data from each mother-offspring pair in relation to each male in the population. For each such ‘trio’, the program Paternity.matrix.py calculates the log-likelihood of the data first assuming that the male is the sire and then considering it as an unrelated male randomly sampled from the population. The difference between these log-likelihoods are the quantities that populate the ‘paternity matrix’ with mother-offspring pairs as rows and males as columns. This matrix is input to the FAPs program (Ellis et al. 2018) for subsequent inference of the number of offspring sired by each male. While some mother-offspring pairs overwhelmingly favor a particular male as the sire, fractional assignments of paternity were also counted using the sires() function of FAPs. In these calculations, were only able to consider males called for at least 10% of SNPs (n = 481). The paternity analyses were executed by application of the following series of programs: Paternity.matrix.py, loci.per.sire.py, Paternity.hardcalls.py, fap.run.py, and Analyze.faps.output.py.

**D. SNP level tests for differential paternity and female fecundity (seedset)**

We estimate selection through differential paternity by relating the genotype likelihoods (at each SNP for each male) to the estimated number of offspring sired. Without any uncertainty in genotyping, the mean fitness (paternity) estimate for each genotype would simply be the average paternity of plants in each genotype class (RR or RA or AA). With uncertain calls, a weighted average is required:

$W_{RR}=\frac{\sum_{j}^{N_{m}} u_{RR,j}P_{j}}{\sum_{j}^{N_{m}} u_{RR,j}}{;W}_{RA}=\frac{\sum_{j}^{N_{m}} u_{RA,j}P_{j}}{\sum_{j}^{N_{m}} u_{RA,j}};W_{AA}=\frac{\sum_{j}^{N_{m}} u_{AA,j}P_{j}}{\sum_{j}^{N_{m}} u_{AA,j}}$

where $u_{x,j}$ is the posterior probability for genotype x in male plant j, $P_{j}$ is the paternity of that plant, and the sums are taken over all $N_{m}$ males. The posterior probability is simply the genotype likelihood for x multiplied by the Hardy-Weinberg frequency of x standardized to sum to 1 across genotypes. We can derive the within sex predicted change in allele frequency using the standard formula (Hartl and Clark 1989): $\Delta q=\frac{q^{2}W_{RR}+q(1-q)W_{RA}}{\bar{W}}-q$. For females, we use a two stage model to accommodate the large number of zeros (complete failure to set seed) and the highly right-skewed distribution of seed number by those that did (Supplemental Figure S2). We obtain two W values for each genotype: W_1_ is the mean of 0 / 1 based on a plant whether the maternal plant produced any seed, W_2_ is the average Log(seed) among plants setting at least one seed. These are calculated with genotype likelihood weights as above. The overall fitness of each genotype is estimated as W_1_ Exp(W_2_), with $\Delta q$ for female fecundity calculated using these combined values.

The program “Paternity. and.seed.dp.py” executes these calculations, producing $\Delta q$ for both female fecundity and paternity at each SNP. We used permutation to test whether each SNP specific $\Delta q$ differed from zero. The SNP-specific null distribution for each $\Delta q$ was established by permuting the fitness values against genotypes *within* each sex. We then determined the distribution of $\Delta q$ (separately for each sex) across 10,000 such scrambles for each SNP. As expected, the mean $\Delta q$ is invariable close to zero and the overall distributions are very normal (Supplemental Figure S1). The variances differ among SNPs owing to allele frequency and the number/quality of genotyped individuals. However, estimating that variance separately for each SNP, we can test whether $\Delta q$ differs from zero for each particular SNP using the t-distribution and the variance for that SNP.

The analyses (both for significance of individual SNP tests and correlations between tests) were executed using the programs permute.MF.py, DP.SE.permreps.py, and Apply.SE.toreal.dp.py.

**E. Comparing selection through different fitness components**

We aligned results from the selection component tests for each SNP and estimated associations between the five $\Delta q$ values (different components) across the 55,145 SNPs using the Spearman Rank correlation:

| *Spearman ρ* | Male selection | Paternity | Seedset | Male survival |
| --- | --- | --- | --- | --- |
| Paternity | **0.108** |  |  |  |
| Male survival | 0.042 | -0.001 | 0.007 |  |
| Female survival | -0.046 | **-0.099** | 0.017 | 0.046 |

We used permutation to assess significance of apparent associations between components (Spearman ρ ≠ 0). We independently permuted seedset (female fecundity) and survival within females, paternity and survival within males. On each permuted dataset, we recalculated ${\Delta q}_{male viability}$, ${\Delta q}_{female viability}$, ${\Delta q}_{seedset}$, and ${\Delta q}_{paternity}$ for each SNP and aligned these to the ${\Delta q}_{male selection}$ values (unaffected by permutation). We then calculated Spearman’s ρ using all SNPs in the permutation replicate for each pair of $\Delta q$. Permutation preserves inter-locus associations (LD) and thus provides a robust “null distribution”, *i.e.* the range of possible results for ρ across 5000 replicates when $\Delta q$ for each component is determined independently. Comparing values in table above to their respective permutation distributions, two associations proved significant: a positive correlation between male selection and paternity (p < 0.004) and a negative association between paternity and female survival (p < 0.013). These relationships are depicted in Fig 3 of main text. The calculations were performed using Aligned.correlations.v2.py.

**F. Relating male and female fitness measures to phenotypes**

Statistical analyses for sex ratio, survival, sexual dimorphism, and the relationship between flowering traits and fitness were performed using JMP Pro 15 (SAS Institute). Deviation from an equal sex ratio was made with a chi-square test, and the proportion of individuals of each sex that died was compared with a *Z*-test for proportions. Flowering duration (days) and the total number of flowers produced were ln transformed prior to analyses. Comparisons of means for the two sexes were made with a *t*-test. The relationship between survival and fitness was determined with a nominal logistic regression, and the relationship between the flowering traits and fitness was determined with a linear regression. Comparisons between the sexes in the strength of these relationships were not made due to the likelihood of differences in the certainly of fitness measures in males compared to females (Morrissey 2016).

**G. Estimating linkage disequilibria**

Associations between alleles at different SNPs can be estimated from the covariance of diploid genotype scores (Rogers and Huff 2009). From this covariance, we can calculate the familiar squared correlation coefficient (r^2^) routinely used as a measure of LD. In calculating r^2^, we only considered a genotype call if the posterior probability for the most probable genotype was > 0.95. We only considered a pair of SNPs if both loci were called in at least 20 individuals. For SNPs identified in selection analyses, we calculated all pairwise r^2^ using the program LD.among.SigSNPs.py. We then clustered SNPs into loci using LD.classifier.py. To determine LD patterns broadly, we randomly selected 1000 SNPs. For each, we estimated r^2^ with all other SNPs in the genome using the program LD1.py. We compiled the outputs from these 1000 SNPs to produce Supplemental Figure S4 using the program compile.LD1.py.

Refs

Catchen, J., P. A. Hohenlohe, S. Bassham, A. Amores, and W. A. Cresko. 2013. Stacks: an analysis tool set for population genomics. Molecular ecology 22:3124-3140.

Ellis, T. J., D. L. Field, and N. H. Barton. 2018. Efficient inference of paternity and sibship inference given known maternity via hierarchical clustering. Molecular Ecology Resources 18:988-999.

Hartl, D. L. and A. G. Clark. 1989. Principles of population genetics. Sinauer associates, Sunderland, Massachusetts.

Li, H. and R. Durbin. 2009. Fast and accurate short read alignment with Burrows-Wheeler Transform. Bioinformatics 25:1754–1760.

Monnahan, P. J., J. Colicchio, and J. K. Kelly. 2015. A genomic selection component analysis characterizes migration-selection balance. Evolution 69:1713-1727.

Papadopulos, A. S. T., M. Chester, K. Ridout, and D. A. Filatov. 2015. Rapid Y degeneration and dosage compensation in plant sex chromosomes. Proceedings of the National Academy of Sciences 112:13021-13026.

Rogers, A. R. and C. Huff. 2009. Linkage Disequilibrium Between Loci With Unknown Phase. Genetics 182:839-844.

Virtanen, P., R. Gommers, T. E. Oliphant, M. Haberland, T. Reddy, D. Cournapeau, E. Burovski, P. Peterson, W. Weckesser, J. Bright, S. J. van der Walt, M. Brett, J. Wilson, K. J. Millman, N. Mayorov, A. R. J. Nelson, E. Jones, R. Kern, E. Larson, C. J. Carey, İ. Polat, Y. Feng, E. W. Moore, J. VanderPlas, D. Laxalde, J. Perktold, R. Cimrman, I. Henriksen, E. A. Quintero, C. R. Harris, A. M. Archibald, A. H. Ribeiro, F. Pedregosa, P. van Mulbregt, A. Vijaykumar, A. P. Bardelli, A. Rothberg, A. Hilboll, A. Kloeckner, A. Scopatz, A. Lee, A. Rokem, C. N. Woods, C. Fulton, C. Masson, C. Häggström, C. Fitzgerald, D. A. Nicholson, D. R. Hagen, D. V. Pasechnik, E. Olivetti, E. Martin, E. Wieser, F. Silva, F. Lenders, F. Wilhelm, G. Young, G. A. Price, G.-L. Ingold, G. E. Allen, G. R. Lee, H. Audren, I. Probst, J. P. Dietrich, J. Silterra, J. T. Webber, J. Slavič, J. Nothman, J. Buchner, J. Kulick, J. L. Schönberger, J. V. de Miranda Cardoso, J. Reimer, J. Harrington, J. L. C. Rodríguez, J. Nunez-Iglesias, J. Kuczynski, K. Tritz, M. Thoma, M. Newville, M. Kümmerer, M. Bolingbroke, M. Tartre, M. Pak, N. J. Smith, N. Nowaczyk, N. Shebanov, O. Pavlyk, P. A. Brodtkorb, P. Lee, R. T. McGibbon, R. Feldbauer, S. Lewis, S. Tygier, S. Sievert, S. Vigna, S. Peterson, S. More, T. Pudlik, T. Oshima, T. J. Pingel, T. P. Robitaille, T. Spura, T. R. Jones, T. Cera, T. Leslie, T. Zito, T. Krauss, U. Upadhyay, Y. O. Halchenko, Y. Vázquez-Baeza and C. SciPy. 2020. SciPy 1.0: fundamental algorithms for scientific computing in Python. Nature Methods 17:261-272.

Wessinger, C. A., J. K. Kelly, P. Jiang, M. D. Rausher, and L. C. Hileman. 2018. SNP-skimming: A fast approach to map loci generating quantitative variation in natural populations. Molecular Ecology Resources 18:1402-1414.
