# Supplementary material for: Sex‐specific natural selection on SNPs in Silene latifolia
Source: Evol Lett. 2022 May 27;6(4):308–18. doi: 10.1002/evl3.283 (PMC9346077; doi:10.1002/evl3.283)
Supplement: Supplementary file 2 — Figure S1. We use permutation to establish significance levels for SNPs affecting paternity and seed set. Figure S2. The distribution of seeds per female (top panel, n = 687) and offspring sired per male (lower panel, n = 481) Figure S3. The predicted change in allele frequency (minor base) owing to Male selection is reported for SNP with significant tests (Sig means FDR0.1) Figure S4. The density function for LD among SNPs estimated from 1000 randomly selected SNPs (of the 55,145 total) contrasted all other SNPs. Figure S5. The relationship between heterozygosity and number of called SNPs is depicted for the final genotype calls. [file EVL3-6-308-s001.pdf]

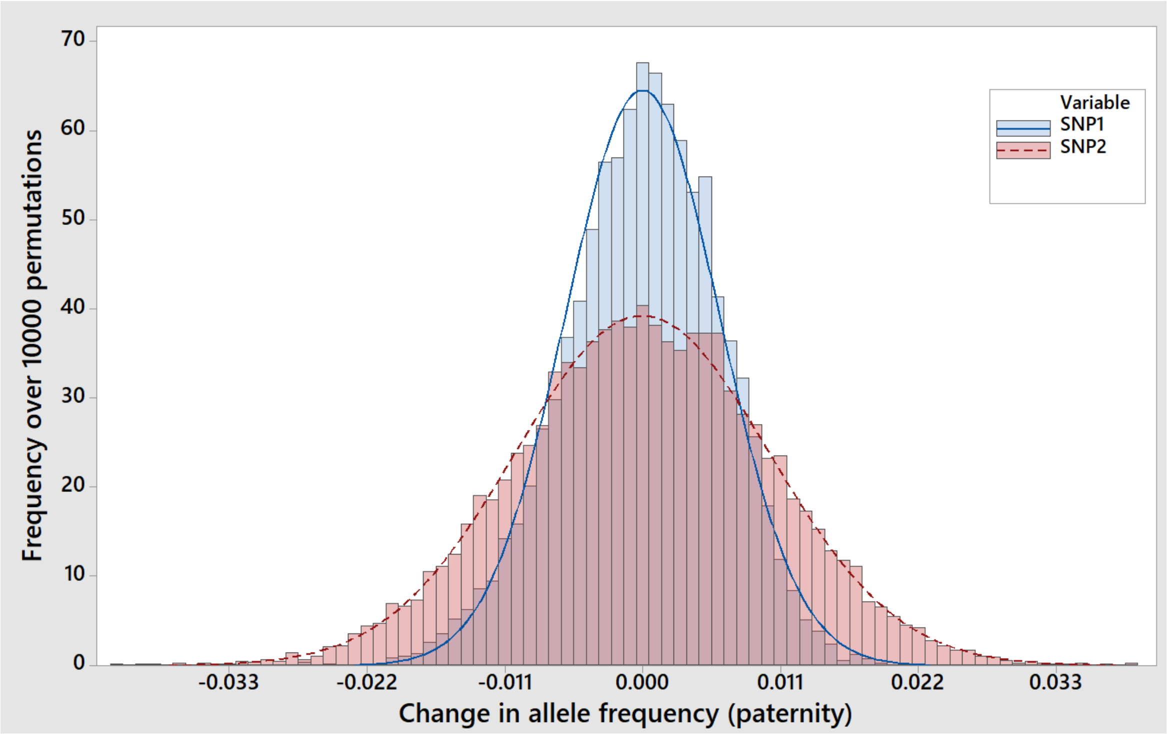

Figure S1. We use permutation to establish significance levels for SNPs affecting paternity and seed set. The null distribution for change in allele frequency is depicted for two SNPs that were significant for paternity selection. The number of genotyped individuals was higher for SNP1 leading to a reduced sampling variance relative to SNP2. Importantly, these distributions are generally normal with a mean of zero.

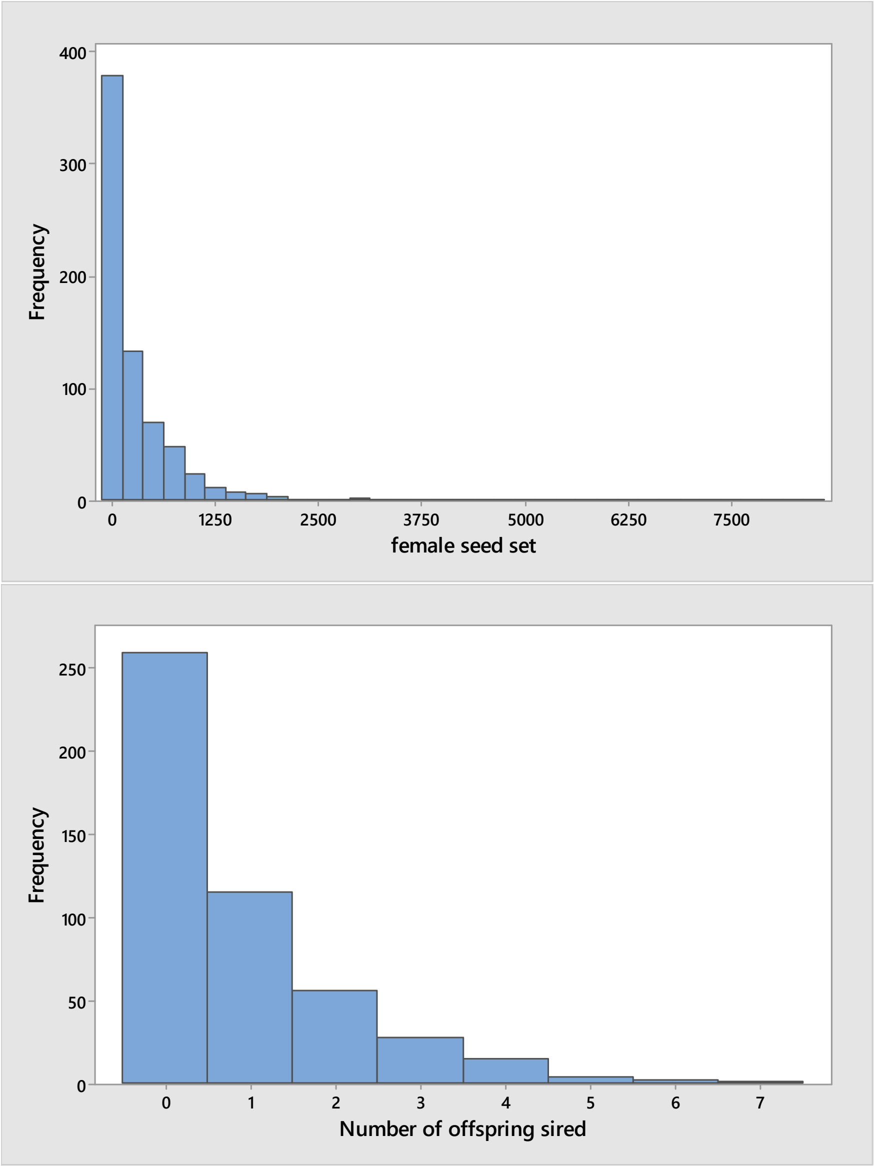

Figure S2. The distribution of seeds per female (top panel, n = 687) and offspring sired per male (lower panel, n = 481).

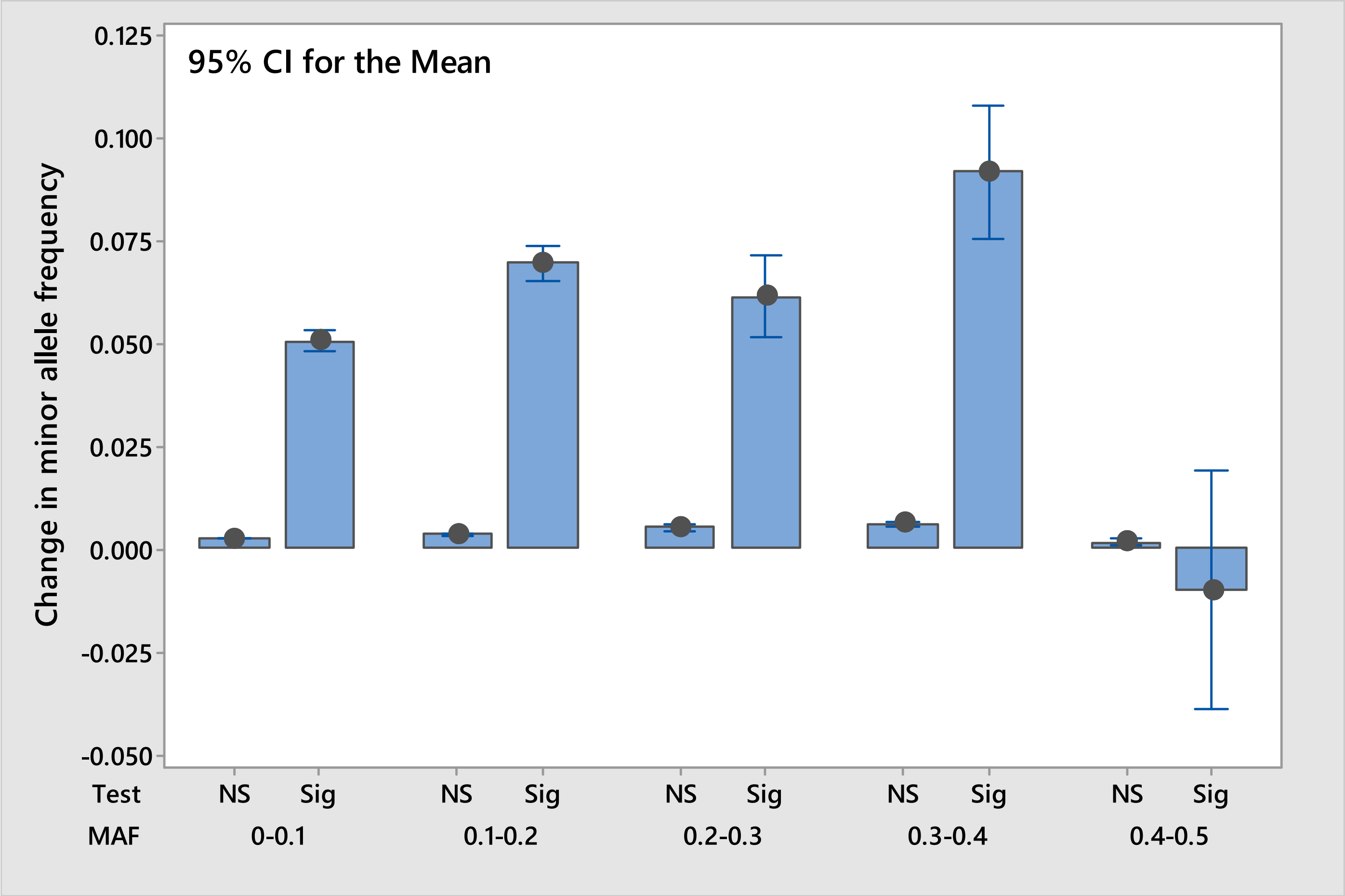

Figure S3. The predicted change in allele frequency (minor base) owing to Male selection is reported for SNP with significant tests (Sig means FDR<0.1) and non-significant tests (NS means p>0.1). Sample sizes: MAF=0-0.1:NS=19662,Sig=604; MAF=0.1-0.2:NS=9775,Sig=545;MAF=0.2-0.3:NS=5641,Sig=218; MAF=0.3-0.4:NS=4392,Sig=93; MAF=0.4-0.5:NS=4264,Sig=58.

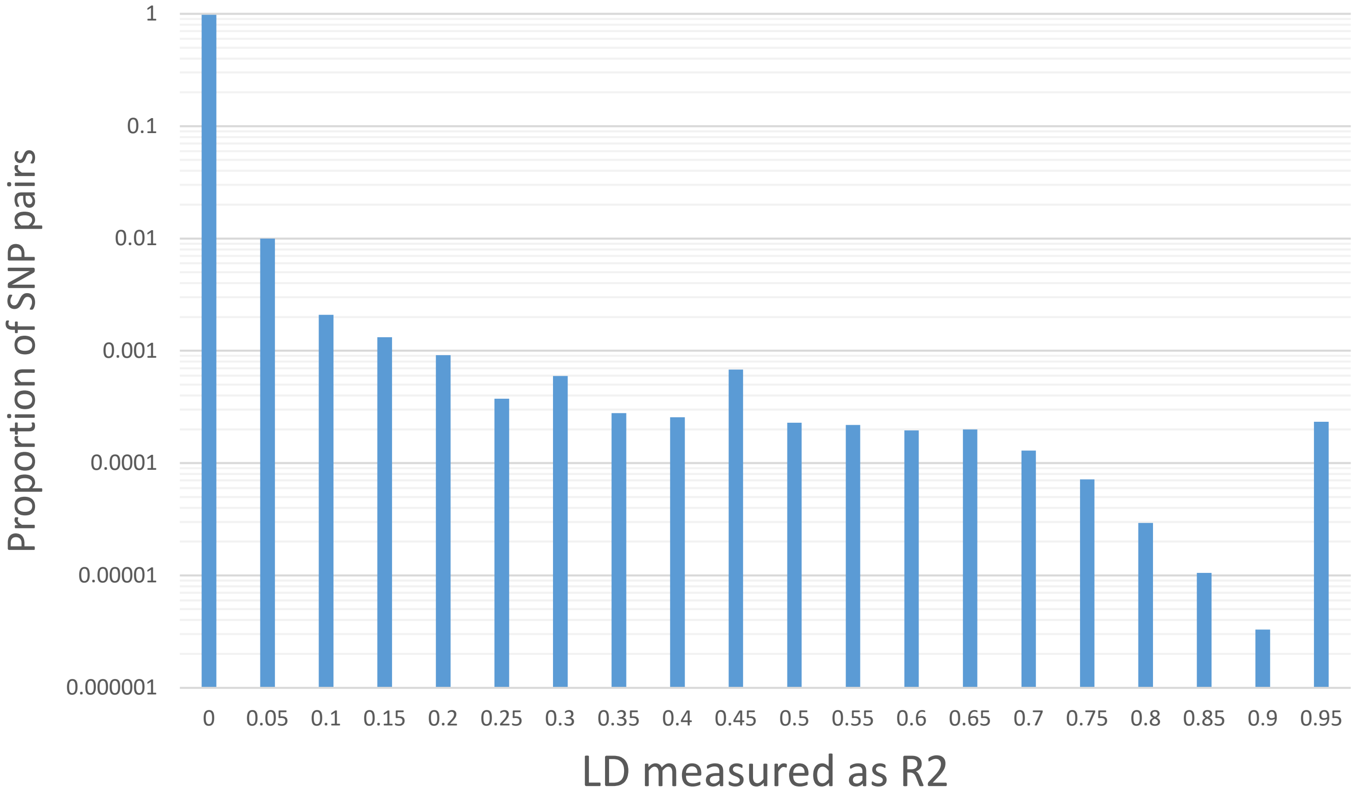

Figure S4. The density function for LD among SNPs estimated from 1000 randomly selected SNPs (of the 55,145 total) contrasted all other SNPs.

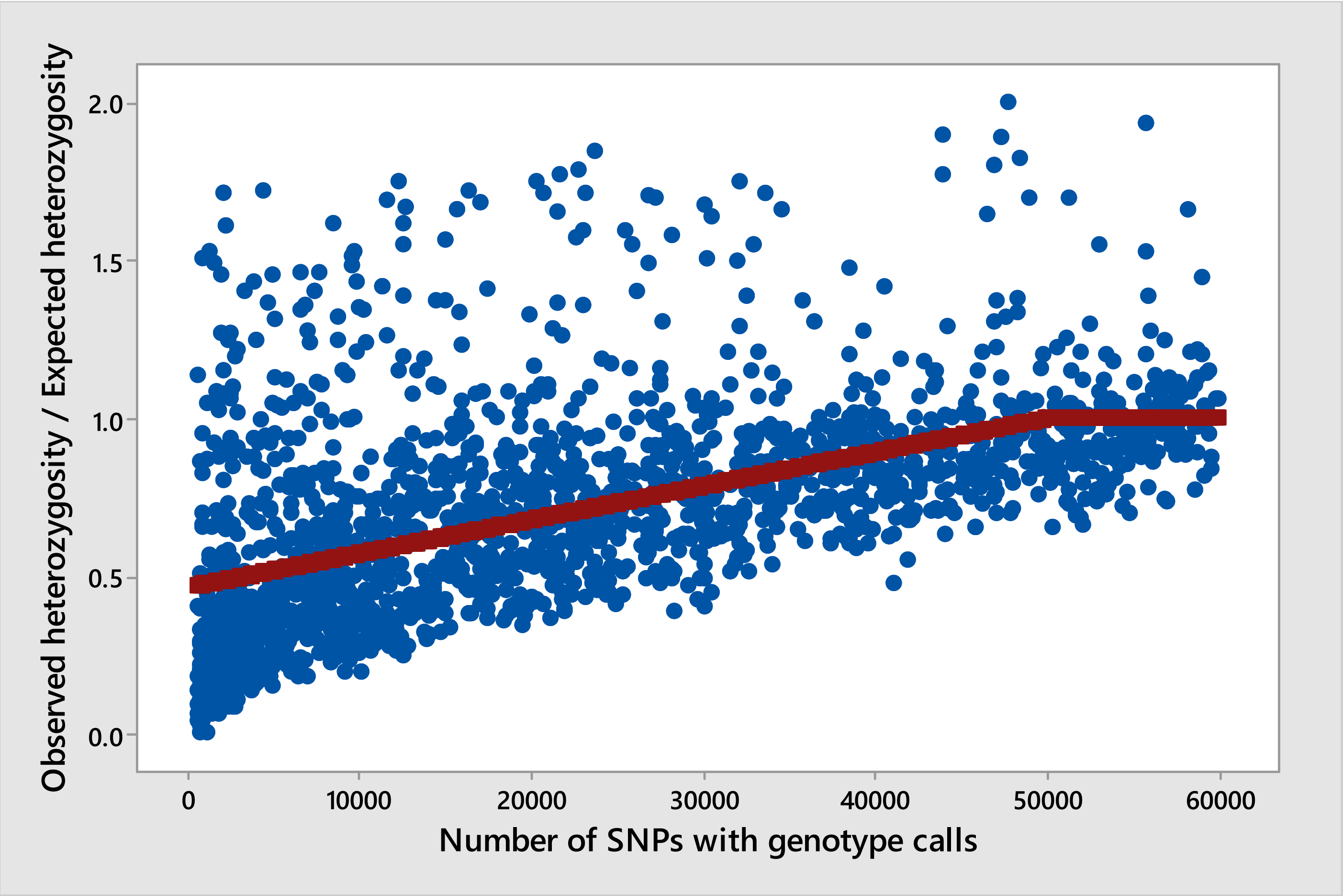

Figure S5. The relationship between heterozygosity and number of called SNPs is depicted for the final genotype calls. The expected heterozygosity for an individual plant is the sum of per-locus values for 2pq at all the SNPs for which it is called. The red line is the estimated probability that a plant that is a heterozygote yields a call to heterozygote in the vcf file (see h parameter in equation S1).
